# Comparison of clinical outcomes in patients with schizophrenia following different long-acting injectable event-driven initiation strategies

**DOI:** 10.1038/s41537-023-00334-3

**Published:** 2023-02-11

**Authors:** Christoph U. Correll, Carmela Benson, Bruno Emond, Charmi Patel, Marie-Hélène Lafeuille, Dee Lin, Laura Morrison, Isabelle Ghelerter, Patrick Lefebvre, Panagiotis Mavros

**Affiliations:** 1 The Zucker Hillside Hospital, Northwell Health, New York, NY USA; 2 Donald and Barbara Zucker School of Medicine at Hofstra/Northwell, Hempstead, NY USA; 3 The Feinstein Institute for Medical Research, Center for Psychiatric Neuroscience, Manhasset, NY USA; 4 Department of Child and Adolescent Psychiatry, Charité Universitätsmedizin, Berlin, Germany; 5 Janssen Scientific Affairs, LLC, Titusville, NJ USA; 6 Analysis Group, Inc., Montréal, QC Canada; 7 KREDHERA, LLC, Hampton, NJ USA

**Keywords:** Schizophrenia, Psychosis

## Abstract

This retrospective study evaluated the benefit of following different long-acting injectable (LAI) initiation strategies based on the timing of behavioral and clinical events among Medicaid beneficiaries with schizophrenia. Adults with schizophrenia initiating oral antipsychotics (OAPs) after 12 months without antipsychotic use or schizophrenia-related inpatient/emergency room (ER) visits (index date) were identified. Patients were categorized into four event-driven LAI initiation strategy cohorts based on observed sequences of behavioral (i.e., OAP adherence) and clinical (i.e., schizophrenia-related inpatient/ER visits) events between index and LAI initiation or censoring—strategy #1: adherent to OAPs without schizophrenia-related inpatient/ER visits; strategy #2: nonadherent to OAPs without schizophrenia-related inpatient/ER visits; strategy #3: one schizophrenia-related inpatient/ER visit; strategy #4: ≥2 schizophrenia-related inpatient/ER visits. Clinical outcomes (i.e., all-cause inpatient/ER visits) were evaluated between OAP initiation and end of follow-up. Comparisons between LAI initiation strategy cohorts were conducted using a dynamic marginal structural model adjusting for baseline characteristics and time-varying confounders. Among 13,444 eligible patients, 13.1%, 53.6%, 15.7%, and 17.6% were following strategies #1–4, respectively; of these, 21.9%, 4.3%, 9.2%, and 6.5% started an LAI (the remaining were censored). Strategy #1 was associated with a greater clinical benefit, with 43%, 69%, and 80% fewer inpatient days (all *p* < 0.05); and 57%, 59%, and 79% fewer ER visits (all *p* < 0.01) vs strategies #2–4, respectively; the clinical benefit was also observed for strategy #2 vs #3–4. Therefore, starting an LAI prior to OAP nonadherence or occurrence of a schizophrenia-related inpatient/ER visit was associated with fewer all-cause inpatient days of inpatient stay and ER visits.

## Introduction

Antipsychotics are the main pharmacologic therapy for patients with schizophrenia^1,2^. Schizophrenia treatment guidelines recommend tailoring pharmacologic intervention based on patient’s disease stage, history of schizophrenia-related relapses, psychiatric and physical comorbidities, response to current or previous antipsychotic(s), potential side effects associated with antipsychotic medication and its tolerability profile, history of antipsychotic medication adherence, transition of care, and patient preference^2–7^.

An important challenge in schizophrenia care is adherence to therapy, which is crucial for reducing the risk of schizophrenia relapse and its negative consequences such as hospitalization and self-harm^8–12^. Studies have demonstrated that long-acting injectable antipsychotics (LAIs), which require less frequent administration than oral antipsychotics (OAPs), are associated with better adherence than OAPs^13–16^. Some studies have also found that treatment with LAIs was associated with significant reductions in healthcare resource utilization, with the magnitude of results varying depending on the study design and patient population^17,18^. Other recognized advantages of LAIs include ensured knowledge about adherence status; lower risk of relapse, hospitalization and mortality; and increased opportunity to receive psychosocial treatments during a state of greater stability^8,14,17^.

Barriers to using LAIs may exist. Surveys conducted among clinicians have identified some reasons underlying the infrequent LAI use, particularly as treatment following first-episode psychosis, including concerns over costs, adverse effects, stigmatization, presumed OAP adherence and nonacceptance of LAI treatment, and clinicians’ perception that LAIs were reserved for later use in the disease course^19–21^.

Despite these barriers, most guidelines recognize the various advantages of LAIs compared to OAPs; however, recommendations for the appropriate timing of LAI initiation with respect to events such as nonadherence and relapse vary considerably across guidelines and will differ based on patient experiences, preferences, and situations^22^. A recent systematic review found that among US guidelines for schizophrenia, recommendations include initiating LAIs as first-line therapy after tolerability and sufficient effectiveness have been assessed with the oral formulation of the same antipsychotic (Florida Medicaid Program)^3^, in patients with history of poor or uncertain adherence (American Psychiatric Association, Harvard South Shore Program)^2,6^, in patients presenting frequent relapses (American Association of Community Psychiatrists [AACP])^23^, after failure of ≥2 OAPs or in nonadherent patients (Florida Medicaid Program, AACP, Oregon Health Authority)^5,23,24^, or when patients prefer the LAI formulation to OAPs (Schizophrenia Patient Outcomes Research Team)^4^.

Given the lack of consensus regarding LAI recommendations, variations in clinical practice, and each patient’s unique journey, there is a need to understand the impact of different LAI treatment initiation strategies on clinical outcomes. More specifically, patients’ start of LAIs may be preceded by clinical events, such as relapses or nonadherence episodes. As such, LAI treatment initiation strategies may be identified based on the observed sequences of previous behavioral and clinical events, resulting in different timing of LAI initiation for each patient. This study therefore aimed to assess clinical outcomes (i.e., inpatient and emergency room [ER] visits) and healthcare costs among Medicaid beneficiaries (from six states) with schizophrenia following different event-driven LAI initiation strategies, identified based on patterns of OAP adherence and schizophrenia-related inpatient admissions or ER visits after initial OAP treatment. To assess the experience of various demographic subgroups on different LAI initiation strategies, analyses were also conducted among patient subgroups based on age, sex at birth, and race. Based on prior studies^25–27^, we hypothesized that starting LAIs while patients are adherent to their current OAP and prior to one or more schizophrenia-related inpatient admissions or ER visits would be associated with significantly better outcomes, and this benefit would be consistent across demographic subgroups.

## Results

### Sample size and strategy cohorts

**Fig. 1:**
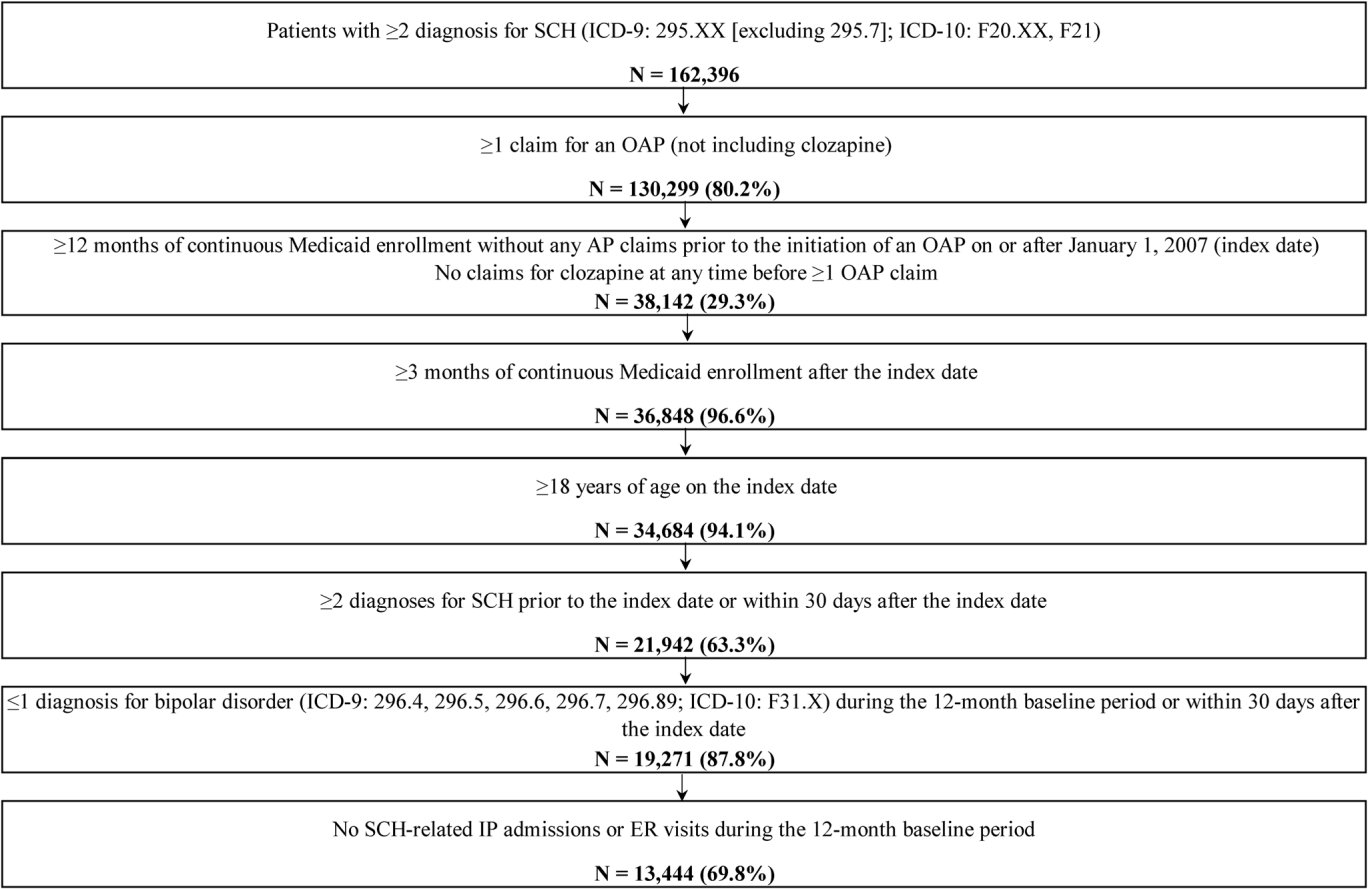
Identification of the study population. AP antipsychotic, ER emergency room, ICD-9/ICD-10 International Classification of Diseases, 9th/10th Revision, IP inpatient, OAP oral antipsychotic, SCH schizophrenia.

**Fig. 2:**
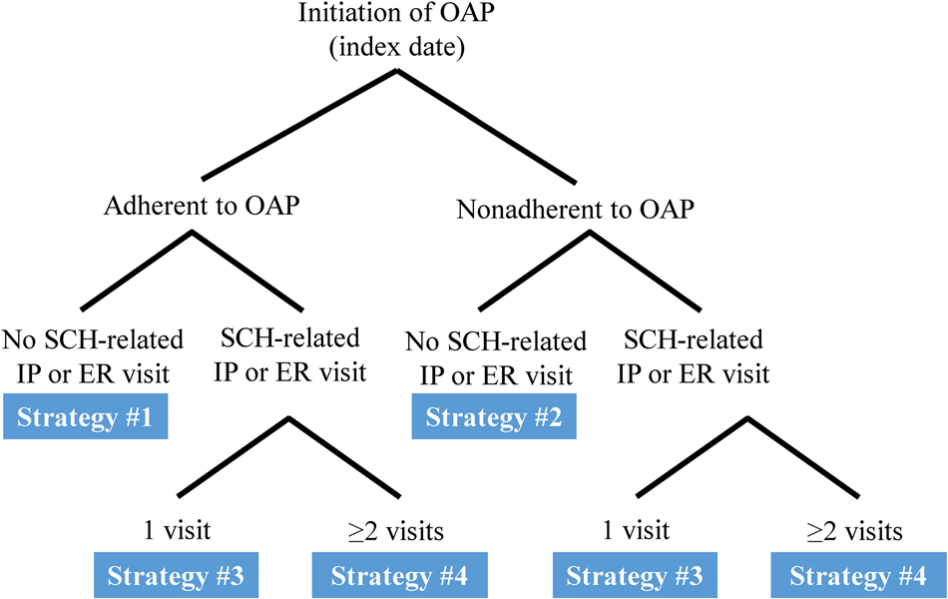
Strategies based on sequence of events. ER emergency room, IP inpatient, OAP oral antipsychotic, SCH schizophrenia.

A total of 13,444 eligible patients were included in this study (Fig. 1). To compare outcomes across different LAI initiation strategies, patients were categorized into four different LAI initiation strategy cohorts based on the observed sequence of behavioral (i.e., OAP adherence) and clinical events (i.e., schizophrenia-related inpatient admissions or ER visits) during the follow-up period (Fig. 2) between index and LAI initiation or censoring. LAI initiation strategy #1 (the most proactive strategy) comprised patients adherent to OAPs without schizophrenia-related inpatient/ER visits; strategy #2 comprised patients nonadherent to OAPs without schizophrenia-related inpatient/ER visits; strategy #3 comprised patients with exactly one schizophrenia-related inpatient/ER visit during the follow-up period; and strategy #4 (the most reactive strategy) comprised patients with ≥2 schizophrenia-related inpatient/ER visits ≥30 days apart (i.e., revolving door patients). At the end of the follow-up period, 1759 (13.1%), 7211 (53.6%), 2111 (15.7%), and 2363 (17.6%) patients were following event-driven LAI initiation strategies #1–4 cohorts, respectively (as defined in Fig. 2).

By the end of the follow-up period, 21.9% of patients in strategy #1 started an LAI and the remaining 78.1% were censored (i.e., patients still at risk of initiating LAI while being adherent to their current OAP and having no schizophrenia-related inpatient admissions or ER visits by the end of the follow-up period). The proportions of patients who started an LAI according to strategies #2–4 were 4.3%, 9.2%, and 6.5%, respectively, with the remaining patients censored (Table 1). This corresponded to an overall 7.8% of patients across cohorts who started an LAI during the follow-up period. By demographic subgroups, this proportion ranged from 6.0% to 11.9% and was higher among patients 18–35 years old (11.9% vs 6.6% in patients >35 years old), males (9.3% vs 6.0% in females), and Blacks (8.6% vs 6.8% in whites/Caucasians). Overall, more patients received a second-generation LAI than a first-generation LAI across all four studied cohorts, but the proportion of patients receiving a first-generation LAI was relatively higher in strategy #1 than in strategies #2–4 (Table 1).

**Table 1:** Characteristics of patients included in each event-driven LAI initiation strategy cohort.

	Treatment strategy #1^a^	Treatment strategy #2^a^	Treatment strategy #3^a^	Treatment strategy #4^a^
	*N* = 1759	*N* = 7211	*N* = 2111	*N* = 2363
Mean follow-up period	3.9 years	4.2 years	5.0 years	6.3 years
Patients initiating an LAI, *n* (%)	386 (21.9%)	308 (4.3%)	195 (9.2%)	154 (6.5%)
Mean time to LAI initiation	0.6 years	1.6 years	2.0 years	4.0 years
First-generation LAI, *n* (%)	189 (49.0%)	107 (34.7%)	70 (35.9%)	47 (30.5%)
Second-generation LAI, *n* (%)	197 (51.0%)	201 (65.3%)	125 (64.1%)	107 (69.5%)
Patients that were censored, *n* (%)	1373 (78.1%)	6903 (95.7%)	1916 (90.8%)	2209 (93.5%)

*ER* emergency room, *IP* inpatient, *LAI* long-acting injectable, *OAP* oral antipsychotic, *SCH* schizophrenia.

^a^Strategy cohorts were determined at transition to LAI or censoring for each patient by considering all information from the index date up until that point. The following definitions were used to categorize patients into event-driven LAI initiation strategies: Strategy #1: Patients with adherence and no SCH-related IP admissions or ER visits between OAP and either LAI initiation or censoring; Strategy #2: Patients with nonadherence and no SCH-related IP admissions or ER visits between OAP and either LAI initiation or censoring; Strategy #3: Patients with exactly one SCH-related IP admission or ER visit between OAP and either LAI initiation or censoring; Strategy #4: Patients with ≥2 SCH-related IP admissions or ER visits ≥30 days apart between OAP and either LAI initiation or censoring.

### Baseline demographic and clinical characteristics

During the 12 months prior to the index date (i.e., before the date of the first OAP claim), across cohorts, mean age was 47.8–48.9 (standard deviation [SD] = 14.0–15.5) years; 39.7–48.2% were female (51.8–60.3% were male); and 27.5–37.7% were Black (Table 2). Patients treated according to strategy #1 had a lower mean baseline Quan-Charlson Comorbidity Index (Quan-CCI) than those treated according to strategies #2–4 (0.7 vs 1.0–1.1, *p* < 0.0001) and had fewer unique mental health diagnoses (2.0 vs 2.3–2.5, *p* < 0.0001). During the baseline period, a lower proportion of patients treated according to strategy #1 had an all-cause inpatient admission than those treated according to strategies #2–4 (13.8% vs 21.8–24.5%, *p* < 0.0001). The same was true for ER visit (25.3% vs 38.4–42.7%, *p* < 0.0001). Meanwhile, patients treated according to strategy #1 had similar baseline all-cause total healthcare costs as those treated according to strategies #2–4 ($1255 vs $1181–$1199, *p* = 0.7981). Key baseline characteristics by demographic subgroup are shown in Supplementary Table 1.

**Table 2:** Baseline characteristics evaluated during the 12-month baseline period.

	Strategy #1^a^	Strategy #2^a^	Strategy #3^a^	Strategy #4^a^	*p*-value^b^
	*N* = 1759	*N* = 7211	*N* = 2111	*N* = 2363	
Age at index date (years), mean ± SD [median]	47.8 ± 14.3 [48.3]	48.9 ± 15.5 [48.8]	48.8 ± 14.9 [49.1]	47.8 ± 14.0 [48.3]	0.0013*
18–35 years, *n* (%)	413 (23.5)	1587 (22.0)	473 (22.4)	500 (21.2)	0.3469
Sex at birth, *n* (%)					
Female	698 (39.7)	3,475 (48.2)	994 (47.1)	1096 (46.4)	<0.0001*
Male	1061 (60.3)	3736 (51.8)	1117 (52.9)	1267 (53.6)	<0.0001*
Race, *n* (%)					
White/Caucasian	981 (55.8)	3601 (49.9)	1128 (53.4)	1205 (51.0)	<0.0001*
Black	484 (27.5)	2600 (36.1)	716 (33.9)	890 (37.7)	<0.0001*
Hispanic	20 (1.1)	44 (0.6)	10 (0.5)	16 (0.7)	0.0607
Other	54 (3.1)	565 (7.8)	130 (6.2)	101 (4.3)	<0.0001*
Unknown	220 (12.5)	401 (5.6)	127 (6.0)	151 (6.4)	<0.0001*
State, *n* (%)					
Missouri	915 (52.0)	2941 (40.8)	920 (43.6)	982 (41.6)	<0.0001*
Wisconsin	356 (20.2)	670 (9.3)	218 (10.3)	252 (10.7)	<0.0001*
New Jersey	159 (9.0)	1822 (25.3)	430 (20.4)	472 (20.0)	<0.0001*
Mississippi	133 (7.6)	781 (10.8)	185 (8.8)	218 (9.2)	<0.0001*
Kansas	113 (6.4)	655 (9.1)	263 (12.5)	341 (14.4)	<0.0001*
Iowa	83 (4.7)	342 (4.7)	95 (4.5)	98 (4.1)	0.6723
Year of index date, *n* (%)					
2007–2010	808 (45.9)	3312 (45.9)	1194 (56.6)	1671 (70.7)	<0.0001*
2011–2014	512 (29.1)	2505 (34.7)	636 (30.1)	540 (22.9)	<0.0001*
2015–2018	439 (25.0)	1394 (19.3)	281 (13.3)	152 (6.4)	<0.0001*
Type of healthcare plan, *n* (%)					
Fee-for-service only	961 (54.6)	3683 (51.1)	1175 (55.7)	1360 (57.6)	<0.0001*
Both managed care and fee-for-service	309 (17.6)	2211 (30.7)	560 (26.5)	601 (25.4)	<0.0001*
Managed care only	308 (17.5)	918 (12.7)	277 (13.1)	269 (11.4)	<0.0001*
No baseline medical claim to confirm plan	181 (10.3)	399 (5.5)	99 (4.7)	133 (5.6)	<0.0001*
Dual Medicaid\Medicare coverage, *n* (%)	752 (42.8)	3488 (48.4)	1109 (52.5)	1283 (54.3)	<0.0001*
Quan-Charlson comorbidity index, mean ± SD [median]	0.7 ± 1.3 [0.0]	1.1 ± 1.8 [0.0]	1.0 ± 1.8 [0.0]	1.0 ± 1.7 [0.0]	<0.0001*
Number of unique mental health diagnoses, mean ± SD [median]	2.0 ± 2.1 [1.0]	2.3 ± 2.3 [2.0]	2.5 ± 2.3 [2.0]	2.5 ± 2.4 [2.0]	<0.0001*
Type of SCH disorder^c^, *n* (%)					
Schizoaffective disorder	430 (24.4)	1641 (22.8)	609 (28.8)	685 (29.0)	<0.0001*
Unspecified SCH	418 (23.8)	1652 (22.9)	548 (26.0)	620 (26.2)	0.0014*
Paranoid type SCH	252 (14.3)	936 (13.0)	359 (17.0)	513 (21.7)	<0.0001*
Prevalent comorbidities, *n* (%)					
Psychoses	1069 (60.8)	4322 (59.9)	1454 (68.9)	1648 (69.7)	<0.0001*
Hypertension	457 (26.0)	2523 (35.0)	723 (34.2)	851 (36.0)	<0.0001*
Depression	340 (19.3)	2054 (28.5)	577 (27.3)	629 (26.6)	<0.0001*
Diabetes	278 (15.8)	1464 (20.3)	446 (21.1)	507 (21.5)	<0.0001*
Substance-related and addictive disorders	224 (12.7)	1344 (18.6)	398 (18.9)	532 (22.5)	<0.0001*
Other mental health-related medication use, *n* (%)					
Antidepressants	418 (23.8)	2156 (29.9)	588 (27.9)	557 (23.6)	<0.0001*
Anxiolytics	349 (19.8)	1718 (23.8)	517 (24.5)	556 (23.5)	0.0022*
Mood stabilizers	268 (15.2)	1170 (16.2)	317 (15.0)	359 (15.2)	0.3941
All-cause, non-SCH-related resource utilization, *n* (%)					
Had ≥1 IP admission	243 (13.8)	1569 (21.8)	467 (22.1)	578 (24.5)	<0.0001*
Had ≥ 1 ER visit	445 (25.3)	2766 (38.4)	827 (39.2)	1,010 (42.7)	<0.0001*
All-cause total healthcare costs PPPM (US $2019), mean ± SD [median]	1255 ± 2827 [254]	1199 ± 2564 [268]	1190 ± 2342 [325]	1181 ± 2256 [340]	0.7981
Pharmacy costs	104 ± 498 [2]	106 ± 410 [5]	96 ± 356 [3]	86 ± 341 [6]	0.1923
Medical costs	1151 ± 2739 [208]	1093 ± 2484 [202]	1095 ± 2261 [252]	1095 ± 2183 [285]	0.8371

*CCI* Charlson comorbidity index, *ER* emergency room, *ICD-9/ICD-10* International Classification of Diseases, 9th/10th Revision, *IP* inpatient, *LAI* long-acting injectable, *OAP* oral antipsychotic, *PPPM* per-patient-per-month, *SCH* schizophrenia, *SD* standard deviation, *US* United States.

*Significant at the 5% level.

^a^Strategy cohorts were determined at transition to LAI or censoring for each patient by considering all information from the index date up until that point. The following definitions were used to categorize patients into event-driven LAI initiation strategies: Strategy #1: Patients with adherence and no SCH-related IP admissions or ER visits between OAP and either LAI initiation or censoring; Strategy #2: Patients with nonadherence and no SCH-related IP admissions or ER visits between OAP and either LAI initiation or censoring; Strategy #3: Patients with exactly one SCH-related IP admission or ER visit between OAP and either LAI initiation or censoring; Strategy #4: Patients with ≥2 SCH-related IP admissions or ER visits ≥30 days apart between OAP and either LAI initiation or censoring.

^b^*P*-values were calculated using analysis of variance models for continuous variables and chi-square tests for categorical variables.

^c^Types of SCH disorder were identified based on the first 4 digits of the ICD-9/ICD-10 codes for schizophrenia diagnosis. Types of SCH disorder are not mutually exclusive.

### Clinical outcomes

**Fig. 3:**
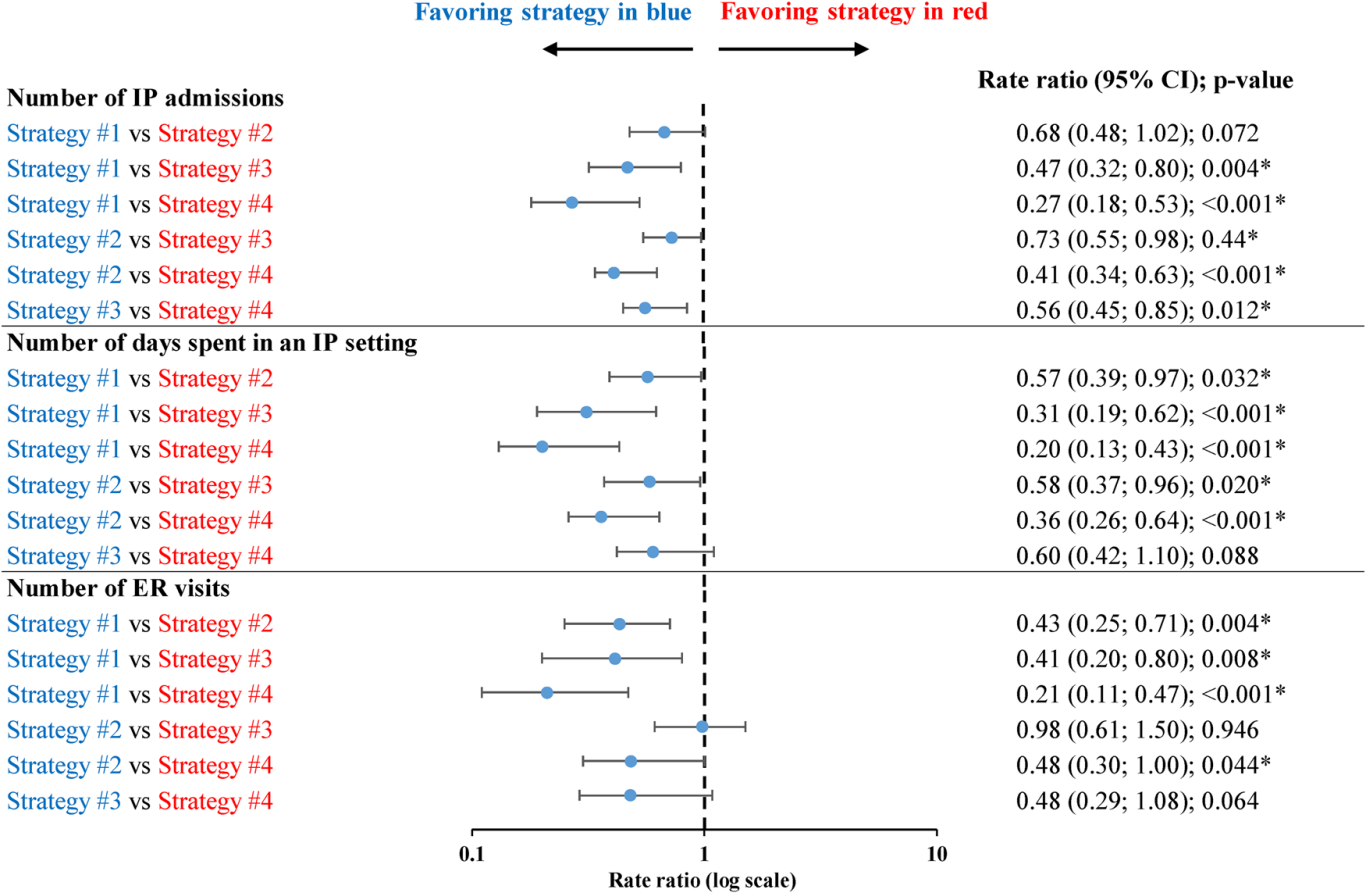
Comparison of clinical benefit evaluated during the follow-up period between event-driven LAI initiation strategies^a,b^. CI confidence interval, ER emergency room, IP inpatient, LAI long-acting injectable, OAP oral antipsychotic, SCH schizophrenia. *Significant at the 5% level. ^a^Clinical benefit was measured by weighted all-cause IP admissions, IP stays, and ER visits per-patient-per-month. ^b^Strategy cohorts were determined at transition to LAI or censoring for each patient by considering all information from the index date up until that point. The following definitions were used to categorize patients into event-driven LAI initiation strategies: Strategy #1: Patients with adherence and no SCH-related IP admissions or ER visits between OAP and either LAI initiation or censoring; Strategy #2: Patients with nonadherence and no SCH-related IP admissions or ER visits between OAP and either LAI initiation or censoring; Strategy #3: Patients with exactly one SCH-related IP admission or ER visit between OAP and either LAI initiation or censoring; Strategy #4: Patients with ≥2 SCH-related IP admissions or ER visits ≥30 days apart between OAP and either LAI initiation or censoring.

Beginning from the time the patients initiated OAP through the end of the follow-up period, including the period when patients transitioned to LAI, and after adjusting for baseline patient characteristics and time-varying confounders, starting an LAI prior to evidence of nonadherence or the occurrence of a schizophrenia-related inpatient/ER visit (strategy #1) was associated with greater clinical benefit (i.e., fewer inpatient admissions, days of inpatient stay, and ER visits) than initiating an LAI subsequent to nonadherence or schizophrenia-related inpatient/ER visit(s) (strategies #2–4). Specifically, strategy #1 was associated with 32% (*p* = 0.072), 53% (*p* = 0.004), and 73% (*p* < 0.001) fewer inpatient admissions; 43%, 69%, and 80% fewer days of inpatient stay (all *p* < 0.05); and 57%, 59%, and 79% fewer ER visits (all *p* < 0.01) during the follow-up period relative to strategy #2, #3, or #4, respectively (Fig. 3). Strategy #2 was associated with 27% and 59% fewer inpatient admissions (all *p* < 0.05), 42% and 64% fewer days of inpatient stay (all *p* < 0.05), and 2% (*p* = 0.946) and 52% (*p* < 0.05) fewer ER visits relative to strategy #3 or #4, respectively. Among demographic subgroups, similar reductions in inpatient admissions, days of inpatient stay, and ER visits were observed for strategies #1–2 relative to strategies #3–4 during the follow-up period (Supplementary Table 2).

### Cost outcomes

Compared to strategies #3–4, strategies #1–2 had lower or similar mean all-cause medical costs during the follow-up period, which resulted in lower or similar mean all-cause total healthcare costs (Table 3). Specifically, while strategy #1 was associated with higher mean monthly total healthcare costs than strategy #2, it was associated with numerically lower mean monthly total healthcare costs than strategies #3–4 (cost difference range: −$109 to −$227, *p* ≥ 0.05). Meanwhile, strategy #2 was associated with significantly lower mean monthly total healthcare costs than strategies #3–4 (−$494 to −$651, *p* < 0.05); the cost difference was mainly driven by a reduction in medical costs.

**Table 3:** Comparison of all-cause weighted healthcare costs PPPM evaluated during the follow-up period between event-driven LAI initiation strategies^a^.

	Mean monthly cost difference (per patient), 2019 USD (95% CI); *p*-value					
	Strategy #1 vs #2	Strategy #1 vs #3	Strategy #1 vs #4	Strategy #2 vs #3	Strategy #2 vs #4	Strategy #3 vs #4
Total costs	434 (134; 953); <0.001*	−109 (−451; 440); 0.926	−227 (−647; 432); 0.681	−494 (−801; −133); 0.008*	−651 (−1126; −193); 0.008*	−161 (−612; 270); 0.541
Pharmacy costs	206 (146; 350); <0.001*	207 (133; 343); <0.001*	164 (17; 300); 0.032*	−2 (−73; 37); 0.741	−51 (−240; 14); 0.108	−50 (−230; 42); 0.236
Medical costs	228 (−76; 680); 0.152	−316 (−655; 201); 0.377	−391 (−774; 209); 0.273	−492 (−780; −118); 0.008*	−600 (−1003; −184); 0.008*	−111 (−492; 287); 0.665
IP admissions	3 (−27; 54); 0.717	−94 (−150; −17); 0.016*	−195 (−281; −68); 0.004*	−98 (−154; −37); <0.001*	−201 (−303; −92); <0.001*	−102 (−209; 5); 0.064
ER visits	0 (−6; 9); 0.850	−5 (−19; 5); 0.216	−25 (−47; -3); 0.028*	−5 (−19; 2); 0.216	−25 (−46; −5); 0.004*	−21 (−40; 1); 0.072
OP visits	20 (−14; 62); 0.216	29 (−0; 64); 0.056	9 (−31; 42); 0.649	12 (−11; 37); 0.297	−5 (−41; 20); 0.565	−17 (−49; 9); 0.180
LTC admissions	120 (−103; 505); 0.393	−211 (−490; 182); 0.273	−168 (−480; 250); 0.525	−272 (−497; −52); 0.016*	−234 (−498; 14); 0.080	33 (−269; 363); 0.733
Other medical services^b^	85 (−159; 424); 0.381	−35 (−275; 348); 0.826	−13 (−258; 298); 0.998	−130 (−365; 128); 0.365	−135 (−358; 54); 0.212	−3 (−263; 243); 0.854

*CI* confidence interval, *ER* emergency room, *IP* inpatient, *LAI* long-acting injectable, *LTC* long-term care, *OAP* oral antipsychotic, *OP* outpatient, *PPPM* per-patient-per-month, *SCH* schizophrenia, *USD* United States dollars.

*Significant at the 5% level.

^a^Strategy cohorts were determined at transition to LAI or censoring for each patient by considering all information from the index date up until that point. The following definitions were used to categorize patients into event-driven LAI initiation strategies: Strategy #1: Patients with adherence and no SCH-related IP admissions or ER visits between OAP and either LAI initiation or censoring; Strategy #2: Patients with nonadherence and no SCH-related IP admissions or ER visits between OAP and either LAI initiation or censoring; Strategy #3: Patients with exactly one SCH-related IP admission or ER visit between OAP and either LAI initiation or censoring; Strategy #4: Patients with ≥2 SCH-related IP admissions or ER visits ≥30 days apart between OAP and either LAI initiation or censoring.

^b^Other costs include costs for mental-health institute admissions, one-day mental-health institute outpatient visits, home care, and other costs.

## Discussion

This retrospective longitudinal cohort study found that among 13,444 eligible Medicaid beneficiaries in six states, the strategy of starting LAI prior to OAP nonadherence and a schizophrenia-related inpatient/ER visit (most proactive strategy) was associated with greater clinical benefits compared to starting LAI after nonadherence to OAP or experiencing a schizophrenia-related inpatient/ER visits (reactive strategies). This benefit included a reduction in the number of all-cause inpatient admissions and/or days of inpatient stay, and ER visits. This could potentially be explained by patients’ lower disease severity when they are still adherent or have not gone through periods of distress resulting in (one or multiple) schizophrenia-related inpatient/ER visits; for example, it has been shown that antipsychotic nonadherence is associated with greater severity of symptoms^28^. Moreover, results across all demographic subgroups similarly showed that LAI initiation prior to evidence of nonadherence and having a schizophrenia-related inpatient/ER visit was associated with greater clinical benefits than waiting to start LAI after one of these important clinical events.

Current schizophrenia treatment guideline recommendations vary considerably regarding LAI use^2–7,24^. Based on a systematic review of 19 schizophrenia guidelines worldwide, only one guideline recommends LAI use independent of OAP adherence to prevent future nonadherence-related relapses^3^. Ten schizophrenia practice guidelines suggest initiating LAI in patients with signs of nonadherence independent of, or prior to, adverse schizophrenia-related clinical events^22^. In addition, five clinical practice guidelines recommend LAI use as early as the first schizophrenia episode^22^. The current study found that LAI initiation among patients who are adherent to their current OAP and have not experienced a schizophrenia-related inpatient/ER visit resulted in better clinical outcomes. This is consistent with the Florida Medicaid Program recommendations^3^, which is the only guideline recommending the use of LAI even when patients are still adherent to their OAP^22^. Results of this study should help raise awareness among clinicians and payers about potential benefits associated with LAI initiation prior to OAP nonadherence or the occurrence of adverse schizophrenia-related clinical events and highlight the need to re-examine current schizophrenia treatment guideline recommendations pertaining to LAI initiation in a broader patient population.

The current approach compared event-driven treatment strategies all together in a same model (i.e., the marginal structural model [MSM]). This approach, which has been applied in other therapeutic areas^29–31^, allows the comparison of LAI initiation strategies based on different sequences of events by adjusting for the time-varying confounders during the follow-up period. Previous literature has evaluated the clinical outcomes of initiating LAIs relative to OAPs in different schizophrenia populations, including those who had experienced a relapse event or those aged 18–34 years (used to approximate timing of LAI start). Most studies demonstrated a clinical advantage with initiation of LAIs, which resulted in a neutral effect on costs^8,13,18,32–37^. However, results from these studies cannot inform on the best timing for LAI initiation over a patient’s journey to optimize outcomes. The current analysis addresses this need by further refining the categorization of timing of LAI start and comparing different event-driven LAI initiation strategies all together using the same adjusted model.

Overall, the rate of LAI use during the follow-up period was only about 7.8%, which was slightly lower than the national average of 13% recently reported by Patel et al. for the overall Medicaid population across 45 states in 2018^38^. However, based on the Patel et al. study, rates of LAI use varied across the 45 states (4–26%), with the reported rate in the current study within this range; this may account for the difference in the overall rate found in the current study based on only six states. Furthermore, because the first qualifying index date (based on the inclusion/exclusion criteria) starting on 1 January 2007 was used for the study, a large proportion of patients initiated treatment in earlier years (2007–2010), when fewer LAI options were available, and this may also explain the difference between the proportion of LAI users found in the current study and data from Patel et al. The current study also found that the proportion of patients who started an LAI was higher among those 18–35 years old, male, and Black. Future studies are warranted to further evaluate health disparities related to LAI utilization and associated outcomes in these subgroups. Lastly, it should be noted that first-generation LAIs were more used in strategy #1 and second-generation LAIs were more used in other strategies. As some second-generation LAIs are recommended or approved by the Food and Drug Administration for patients with schizoaffective disorder^7,39^, the variations in first- vs second-generation LAI use may be partially due to the higher proportion of patients with schizoaffective disorder in patients treated according to strategies #3–4 than strategy #1 in the current study. In addition, given the higher proportion of patients with dual Medicaid/Medicare coverage in strategies #2–4, second-generation LAIs may have been more used by patients in these strategies than those in strategy #1, because the additional Medicare coverage results in reduced out-of-pocket spending and fewer cost-related barriers^40^. On the other hand, as the proportion of patients with dual Medicaid/Medicare coverage was lower in strategy #1, these patients may have opted for first-generation LAIs because of their lower price. However, future studies should be conducted to further investigate this finding.

The overall low rate of LAI use in the current study is in line with previous reports that LAIs are underused in US clinical practice^41,42^, and this contradicts the documented effectiveness of LAIs in schizophrenia^14,43,44^. Indeed, LAI use for first-episode or newly diagnosed schizophrenia has been associated with reduced relapse rates^27^, better adherence^37^, and increased patient satisfaction relative to OAPs^45^. In addition, a retrospective cohort study showed that LAI initiation ≤1 year after the first recorded schizophrenia diagnosis resulted in lower hospitalization rates and healthcare costs compared with later initiators^46^.

Collectively, the above literature evidence indicates that LAI use early in the disease course has the potential to improve treatment outcomes. The current study expanded this existing literature by demonstrating the greater clinical benefit associated with LAI initiation prior to OAP nonadherence and the occurrence of a schizophrenia-related clinical event, and that such clinical benefit translated into a neutral effect on or reduction in healthcare costs.

Some limitations should be considered when interpreting the results of the current study. First, this study included beneficiaries with Medicaid coverage from six states only, which could limit the generalizability of findings. Second, we assumed claims for OAPs were taken as prescribed, which may lead to a potential overestimation of OAP adherence. Misclassification of the timing of LAI initiation strategy was also possible since antipsychotics given via patient sample programs were not recorded in claims. Third, the onset date of schizophrenia could not be confirmed in the claims; hence, the duration of the disease could not be assessed and adjusted for in the analyses and patients in this study could have included a mix of new antipsychotic users and patients re-starting antipsychotic treatment that may or may not be patients with recent-onset schizophrenia. Fourth, the current categorization of cohorts for strategies #3–4 was based on schizophrenia-related clinical events and patients could have been either OAP-adherent and OAP-nonadherent patients; therefore, these two strategies may be confounded by patient’s adherence status. Likewise, the time to LAI initiation for each cohort in this study was driven by study design. As a longer follow-up time would be needed for each additional behavioral or clinical event to occur prior to LAI initiation, strategy cohorts that needed patients to meet requirements for more events would likely have a prolonged time between the index date and LAI initiation; this is reflected by the increasing mean time to LAI initiation from strategies #1 to #4. Fifth, patients were selected based on the earliest qualifying index date, resulting in more index dates in the earlier years of data availability (2007–2010). Sixth, this study may have been subject to residual confounding due to unmeasured confounders. In addition, while many confounders were adjusted for in the MSM analysis to balance cohorts, the number of confounders used was limited by sample size (particularly the number of patients starting LAI) and confounders that did not change over time or had unusual patterns over time resulted in unstable weights; therefore, to avoid extreme weights, extremely small and large weights were trimmed. To further mitigate this limitation, doubly robust adjustment was made to incorporate characteristics that could not be balanced using MSM.

In conclusion, starting an LAI prior earlier—that is before there is any evidence of nonadherence to current OAP treatment and prior to any adverse schizophrenia-related clinical events (inpatient admission/ER events)—was associated with the greatest clinical benefit, as evidenced by significantly fewer all-cause inpatient admissions, days of inpatient stay, and ER visits. The second most optimal event-driven LAI initiation strategy was after evidence of OAP nonadherence but prior to any adverse schizophrenia-related clinical events. The least favorable strategy was starting LAI after ≥2 schizophrenia-related inpatient/ER visits. Results were consistent across demographic groups, suggesting that starting LAI prior to nonadherence or schizophrenia-related inpatient/ER visits regardless of the patient age, race, or sex can optimize patient outcomes. The findings of this study may inform clinical decision-making and future schizophrenia treatment guidelines pertaining to timing of initiating LAI in patients diagnosed with schizophrenia.

## Methods

### Data source

Medicaid data from Iowa (2006Q1-2017Q1), Kansas (2006Q1-2019Q1), Mississippi (2006Q1-2019Q1), Missouri (2006Q1-2019Q1), New Jersey (2006Q1-2014Q1) and Wisconsin (2006Q1-2013Q4) were used. The Medicaid database includes information on enrollment eligibility, physician visits, hospitalizations, long-term care services, prescription drugs, and other services reimbursed by Medicaid. Cost information is pre-rebate and represents the actual amount paid by Medicaid for services rendered. Data are de-identified and comply with the patient requirements of the Health Insurance Portability and Accountability Act; therefore, no reviews by an institutional review board were required.

### Study design

This was a retrospective longitudinal cohort study. The index date was the date of the first OAP claim on or after 1 January 2007 after applying a 12-month washout period without any antipsychotic claims (i.e., no OAPs, short-acting injectables, or LAIs) or schizophrenia-related inpatient admissions or ER visits, to capture new starts and re-starts of OAPs. The baseline period was defined as the 12-month period pre-index (i.e., prior to the date of first OAP claim, same as the washout period) and the follow-up period (which was used to evaluate study outcomes) was defined as the period from the index date until the earliest among the following events: LAI discontinuation (last day before a period >180 days without LAI treatment), the date of the first claim for clozapine, end of continuous eligibility, end of data availability, or death (Supplementary Fig. 1).

### Patient selection criteria

Patients were included if they were ≥18 years of age at the index date; had ≥2 claims with a diagnosis for schizophrenia on different dates any time prior to or within 30 days after the index date (International Classification of Diseases, 9th Revision [ICD-9]: 295.X [excluding 295.7]; ICD-10: F20.X, F21); had ≥1 claim for an OAP on/after 1 January 2007; and had ≥12 and ≥3 months of continuous Medicaid eligibility before and after the index date, respectively. Patients were excluded if they had ≥1 claim for an antipsychotic during the 12-month washout period or for clozapine at any time prior to the index date; had ≥1 schizophrenia-related inpatient admission or ER visit during the 12-month washout period; and had ≥2 claims on different dates with a diagnosis for bipolar disorder (ICD-9: 296.4, 296.5, 296.6, 296.7, 296.89; ICD-10: F31.X) during the baseline period or within 30 days after the index date (Fig. 1).

### Patient cohorts

The different LAI initiation strategy cohorts were determined based on adherence and schizophrenia-related inpatient admissions or ER visits observed after OAP initiation, until LAI initiation or censoring. The LAI initiation strategies can be considered proactive (i.e., switching to LAI while still adherent to current OAP) or reactive (i.e., switching to LAI after experience of nonadherence, or after experience of multiple relapses). LAI initiation strategy #1 comprised patients adherent to OAPs without schizophrenia-related inpatient/ER visits; strategy #2 comprised patients nonadherent to OAPs without schizophrenia-related inpatient/ER visits; strategy #3 comprised patients with exactly one schizophrenia-related inpatient/ER visit; and strategy #4 comprised patients with ≥2 schizophrenia-related inpatient/ER visits ≥30 days apart (Fig. 2). In the analyses, patients not yet initiating an LAI by the end of their follow-up, but still at risk of following a given strategy based on the sequence of events observed, were considered censored. Adherence was defined as having a proportion of days covered (PDC) by antipsychotics ≥80%, measured over the minimum of the period between index and LAI initiation/censoring or the last 12 months. For pharmacy claims, the number of days covered was taken from days of supply information. For medical claims, the number of days covered was imputed based on prescribing information for each LAI.

The proportion of patients initiating LAI and the time to LAI initiation in each cohort are shown in Table 1.

### Study measures and outcomes

Demographic and clinical characteristics were assessed during the 12-month baseline period (i.e., prior to the date of the first OAP claim) and study outcomes were assessed during the follow-up period. Clinical outcomes included the number of inpatient admissions, number of days spent in an inpatient setting, and number of ER visits. Total healthcare costs, including pharmacy, inpatient, ER, outpatient, long-term care, and other medical service costs, were reported per-patient-per-month and expressed in 2019 US dollars using the medical care component of the Consumer Price Index.

### Statistical analysis

Baseline characteristics were summarized using means, SDs, and medians for continuous variables, and frequencies and proportions for categorical variables. Unadjusted comparisons between the four event-driven LAI initiation strategies at the end of the follow-up period were made using analysis of variance models for continuous variables and chi-square tests for categorical variables.

As the different event-driven LAI initiation strategies followed by patients varied over time and depended on baseline characteristics and time-varying confounders that can affect both treatment decisions and outcomes, a dynamic MSM was used to estimate and compare outcomes across LAI initiation strategies in order to generate an unbiased estimate of the effect of each event-driven LAI initiation strategy on the study outcomes^29,30,47,48^. The authors followed the approach described in Neugebauer et al.^29^, which accounts for baseline and time-varying confounding due to change in disease severity over time affecting both treatment decisions and outcomes. Patients who did not initiate LAIs during the follow-up period were retained in the sample and censored at the end of their follow-up. Based on this approach, each outcome model includes MSM weights created to balance patient cohorts based on confounders occurring prior to initiating LAIs (inverse probability of treatment weight; IPTW) and based on the probability of being censored during the follow-up period (inverse probability of censoring weight; IPCW).

Patient IPTWs were calculated based on the estimated probability of initiating an LAI, and patient IPCWs were calculated based on the estimated probability of being uncensored at each 3-month interval of the follow-up period (Supplementary Fig. 1). For each patient, the final MSM weight was calculated as the cumulative product between treatment and censoring weights at each interval, over the entire follow-up period. The IPTW and IPCW models included baseline covariates (age, sex, race, state, year of the index date, type of healthcare plan [one indicator for fee-for-service and one indicator for dual Medicaid/Medicare coverage], Quan-CCI, number of unique mental health conditions, type of schizophrenia disorder [paranoid schizophrenia, schizoaffective disorder, or unspecified schizophrenia], suicide-related and violent behavior–related diagnoses, all-cause inpatient costs, all-cause ER costs, all-cause mental-health institute costs, all-cause home care costs, all-cause pharmacy costs, and use of mental health-related agents [anxiolytics, antidepressants, and mood stabilizers]) and time-varying covariates (number of schizophrenia-related inpatient admissions, ER visits, and outpatient visits [in prior interval] and cumulative number of schizophrenia-related inpatient admissions and ER visits [in all prior intervals]).

Clinical and cost outcomes were compared between event-driven LAI initiation strategies using doubly robust MSM-weighted generalized estimating equations models, additionally adjusting for baseline home care costs and the presence of a diagnosis for developmental disabilities during the baseline period. A Poisson distribution was used to calculate rate ratios for count outcomes. A normal distribution was used to calculate mean differences for continuous outcomes. To account for the overdispersion of count variables and non-normal distribution of cost variables, non-parametric bootstrap procedures were used to generate 95% confidence intervals and 2-sided p-values.

To assess whether results are similar across demographic subgroups, the comparison of clinical outcomes was replicated among the following demographic subgroups identified in the database: young adults 18–35 years old (to approximate a population of patients recently diagnosed with schizophrenia), patients >35 years old, males, females, whites/Caucasians, and Blacks. MSM weights were re-estimated for each subgroup.

## Supplementary information

Supplementary Materials

## Data Availability

The source data that support the findings of this study were obtained from the individual states pursuant to a data use agreement with each state. Therefore, access to the source data is available only through requests made directly to the corresponding states and not to the authors of this study, being subject to each state’s requirements for data access.

## Code availability

All analyses were conducted using SAS Enterprise Guide 7.1 (SAS Institute, Cary, NC). The SAS programs are proprietary materials of Analysis Group, Inc.; therefore, restrictions apply to the access of these codes, which cannot be made available publicly.
