# Supplementary material for: Comparison of clinical outcomes in patients with schizophrenia following different long-acting injectable event-driven initiation strategies
Source: Schizophrenia (Heidelb). 2023 Feb 11;9(1):9. doi: 10.1038/s41537-023-00334-3 (PMC9922270; doi:10.1038/s41537-023-00334-3)
Supplement: Supplementary file 1 — Supplementary Materials [file 41537_2023_334_MOESM1_ESM.doc]

**Supplementary Figure 1. Study design scheme**

AP = antipsychotic; ER = emergency room; IP = inpatient; LAI = long-acting injectable; MSM = marginal structural model; OAP = oral antipsychotic; SCH = schizophrenia.

**Supplementary Table 1. Baseline characteristics evaluated during the 12-month baseline period among demographic subgroups**

|  | **Strategy #1^1^** |  | **Strategy #2^1^** |  | **Strategy #3^1^** |  | **Strategy #4^1^** |
| --- | --- | --- | --- | --- | --- | --- | --- |
|  | **N = 1,759** |  | **N = 7,211** |  | **N = 2,111** |  | **N = 2,363** |
| **18-35 years old subgroup, n (%)** | **413 (23.5)** |  | **1,587 (22.0)** |  | **473 (22.4)** |  | **500 (21.2)** |
| Age at index date (years), mean ± SD [median] | 29.2 ± 4.5 [29.7] |  | 28.5 ± 4.8 [28.9] |  | 28.5 ± 4.8 [29.0] |  | 28.1 ± 4.7 [28.3] |
| Sex at birth, n (%) |  |  |  |  |  |  |  |
| Female | 102 (24.7) |  | 600 (37.8) |  | 174 (36.8) |  | 193 (38.6) |
| Male | 311 (75.3) |  | 987 (62.2) |  | 299 (63.2) |  | 307 (61.4) |
| Race, n (%) |  |  |  |  |  |  |  |
| White/Caucasian | 200 (48.4) |  | 667 (42.0) |  | 221 (46.7) |  | 206 (41.2) |
| Black | 139 (33.7) |  | 651 (41.0) |  | 172 (36.4) |  | 219 (43.8) |
| Other | 74 (17.9) |  | 269 (17.0) |  | 80 (16.9) |  | 75 (15.0) |
| Type of healthcare plan^2^, n (%) |  |  |  |  |  |  |  |
| Fee-for-service only | 219 (53.0) |  | 707 (44.5) |  | 224 (47.4) |  | 263 (52.6) |
| Managed care only | 93 (22.5) |  | 297 (18.7) |  | 91 (19.2) |  | 75 (15.0) |
| Both managed care and fee-for-service | 61 (14.8) |  | 488 (30.7) |  | 132 (27.9) |  | 125 (25.0) |
| No baseline medical claim to confirm plan | 40 (9.7) |  | 95 (6.0) |  | 26 (5.5) |  | 37 (7.4) |
| Dual Medicaid\Medicare coverage^2^, n (%) | 118 (28.6) |  | 482 (30.4) |  | 145 (30.7) |  | 169 (33.8) |
| Quan-CCI, mean ± SD [median] | 0.2 ± 0.6 [0.0] |  | 0.4 ± 0.9 [0.0] |  | 0.3 ± 0.9 [0.0] |  | 0.4 ± 1.1 [0.0] |
| All-cause total costs, 2019 USD, mean ± SD [median] | 1,146 ± 3,139 [154] |  | 922 ± 2,471 [177] |  | 844 ± 1,966 [191] |  | 1,036 ± 2,679 [190] |
|  |  |  |  |  |  |  |  |
| **>35 years old subgroup, n (%)** | **1,346 (76.5)** |  | **5,624 (78.0)** |  | **1,638 (77.6)** |  | **1,863 (78.8)** |
| Age at index date (years), mean ± SD [median] | 53.5 ± 11.1 [51.5] |  | 54.7 ± 12.2 [52.7] |  | 54.7 ± 11.2 [53.3] |  | 53.1 ± 10.6 [51.4] |
| Sex at birth, n (%) |  |  |  |  |  |  |  |
| Female | 596 (44.3) |  | 2,875 (51.1) |  | 820 (50.1) |  | 903 (48.5) |
| Male | 750 (55.7) |  | 2,749 (48.9) |  | 818 (49.9) |  | 960 (51.5) |
| Race, n (%) |  |  |  |  |  |  |  |
| White/Caucasian | 781 (58.0) |  | 2,934 (52.2) |  | 907 (55.4) |  | 999 (53.6) |
| Black | 345 (25.6) |  | 1,949 (34.7) |  | 544 (33.2) |  | 671 (36.0) |
| Other | 220 (16.3) |  | 741 (13.2) |  | 187 (11.4) |  | 193 (10.4) |
| Type of healthcare plan^2^, n (%) |  |  |  |  |  |  |  |
| Fee-for-service only | 742 (55.1) |  | 2,976 (52.9) |  | 951 (58.1) |  | 1,097 (58.9) |
| Managed care only | 248 (18.4) |  | 1,723 (30.6) |  | 428 (26.1) |  | 476 (25.6) |
| Both managed care and fee-for-service | 215 (16.0) |  | 621 (11.0) |  | 186 (11.4) |  | 194 (10.4) |
| No baseline medical claim to confirm plan | 141 (10.5) |  | 304 (5.4) |  | 73 (4.5) |  | 96 (5.2) |
| Dual Medicaid\Medicare coverage^2^, n (%) | 634 (47.1) |  | 3,006 (53.4) |  | 964 (58.9) |  | 1,114 (59.8) |
| Quan-CCI, mean ± SD [median] | 0.8 ± 1.4 [0.0] |  | 1.3 ± 1.9 [1.0] |  | 1.3 ± 2.0 [0.0] |  | 1.2 ± 1.8 [1.0] |
| All-cause total costs, 2019 USD, mean ± SD [median] | 1,289 ± 2,724 [301] |  | 1,277 ± 2,585 [313] |  | 1,291 ± 2,431 [372] |  | 1,220 ± 2,128 [393] |
|  |  |  |  |  |  |  |  |
| **White/Caucasian subgroup, n (%)** | **981 (55.8)** |  | **3,601 (49.9)** |  | **1,128 (53.4)** |  | **1,205 (51.0)** |
| Age at index date (years), mean ± SD [median] | 50.1 ± 15.0 [50.0] |  | 51.5 ± 16.1 [50.9] |  | 50.9 ± 15.2 [51.6] |  | 50.3 ± 14.2 [50.5] |
| Sex at birth, n (%) |  |  |  |  |  |  |  |
| Female | 410 (41.8) |  | 1,709 (47.5) |  | 513 (45.5) |  | 540 (44.8) |
| Male | 571 (58.2) |  | 1,892 (52.5) |  | 615 (54.5) |  | 665 (55.2) |
| Type of healthcare plan^2^, n (%) |  |  |  |  |  |  |  |
| Fee-for-service only | 641 (65.3) |  | 2,068 (57.4) |  | 694 (61.5) |  | 753 (62.5) |
| Managed care only | 183 (18.7) |  | 1,036 (28.8) |  | 296 (26.2) |  | 312 (25.9) |
| Both managed care and fee-for-service | 94 (9.6) |  | 355 (9.9) |  | 103 (9.1) |  | 102 (8.5) |
| No baseline medical claim to confirm plan | 63 (6.4) |  | 142 (3.9) |  | 35 (3.1) |  | 38 (3.2) |
| Dual Medicaid\Medicare coverage^2^, n (%) | 553 (56.4) |  | 2,184 (60.6) |  | 746 (66.1) |  | 821 (68.1) |
| Quan-CCI, mean ± SD [median] | 0.8 ± 1.3 [0.0] |  | 1.1 ± 1.7 [0.0] |  | 1.1 ± 1.7 [0.0] |  | 1.1 ± 1.6 [1.0] |
| All-cause total costs, 2019 USD, mean ± SD [median] | 1,575 ± 3,318 [379] |  | 1,494 ± 3,057 [316] |  | 1,383 ± 2,588 [370] |  | 1,411 ± 2,506 [437] |
|  |  |  |  |  |  |  |  |
| **Black subgroup, n (%)** | **484 (27.5)** |  | **2,600 (36.1)** |  | **716 (33.9)** |  | **890 (37.7)** |
| Age at index date (years), mean ± SD [median] | 45.1 ± 13.7 [46.2] |  | 46.4 ± 14.1 [46.9] |  | 46.8 ± 13.8 [47.4] |  | 46.0 ± 13.7 [46.7] |
| Sex at birth, n (%) |  |  |  |  |  |  |  |
| Female | 191 (39.5) |  | 1,292 (49.7) |  | 351 (49.0) |  | 448 (50.3) |
| Male | 293 (60.5) |  | 1,308 (50.3) |  | 365 (51.0) |  | 442 (49.7) |
| Type of healthcare plan^2^, n (%) |  |  |  |  |  |  |  |
| Fee-for-service only | 254 (52.5) |  | 1,301 (50.0) |  | 373 (52.1) |  | 515 (57.9) |
| Managed care only | 102 (21.1) |  | 854 (32.8) |  | 208 (29.1) |  | 243 (27.3) |
| Both managed care and fee-for-service | 70 (14.5) |  | 297 (11.4) |  | 98 (13.7) |  | 78 (8.8) |
| No baseline medical claim to confirm plan | 58 (12.0) |  | 148 (5.7) |  | 37 (5.2) |  | 54 (6.1) |
| Dual Medicaid\Medicare coverage^2^, n (%) | 173 (35.7) |  | 954 (36.7) |  | 279 (39.0) |  | 393 (44.2) |
| Quan-CCI, mean ± SD [median] | 0.6 ± 1.4 [0.0] |  | 1.0 ± 1.9 [0.0] |  | 1.1 ± 2.1 [0.0] |  | 1.1 ± 1.8 [0.0] |
| All-cause total costs, 2019 USD, mean ± SD [median] | 1,130 ± 2,394 [204] |  | 1,012 ± 2,069 [292] |  | 1,077 ± 2,174 [347] |  | 1,068 ± 2,107 [313] |
|  |  |  |  |  |  |  |  |
| **Female subgroup, n (%)** | **698 (39.7)** |  | **3,475 (48.2)** |  | **994 (47.1)** |  | **1,096 (46.4)** |
| Age at index date (years), mean ± SD [median] | 51.7 ± 14.7 [50.7] |  | 51.9 ± 16.2 [51.2] |  | 51.7 ± 15.2 [51.8] |  | 50.1 ± 14.5 [50.2] |
| Race, n (%) |  |  |  |  |  |  |  |
| White/Caucasian | 410 (58.7) |  | 1,709 (49.2) |  | 513 (51.6) |  | 540 (49.3) |
| Black | 191 (27.4) |  | 1,292 (37.2) |  | 351 (35.3) |  | 448 (40.9) |
| Other | 97 (13.9) |  | 474 (13.6) |  | 130 (13.1) |  | 108 (9.9) |
| Type of healthcare plan^2^, n (%) |  |  |  |  |  |  |  |
| Fee-for-service only | 378 (54.2) |  | 1,747 (50.3) |  | 557 (56.0) |  | 611 (55.7) |
| Managed care only | 147 (21.1) |  | 1,160 (33.4) |  | 285 (28.7) |  | 321 (29.3) |
| Both managed care and fee-for-service | 105 (15.0) |  | 401 (11.5) |  | 117 (11.8) |  | 122 (11.1) |
| No baseline medical claim to confirm plan | 68 (9.7) |  | 167 (4.8) |  | 35 (3.5) |  | 42 (3.8) |
| Dual Medicaid\Medicare coverage^2^, n (%) | 290 (41.5) |  | 1,713 (49.3) |  | 539 (54.2) |  | 631 (57.6) |
| Quan-CCI, mean ± SD [median] | 0.8 ± 1.4 [0.0] |  | 1.3 ± 2.0 [1.0] |  | 1.3 ± 1.9 [1.0] |  | 1.2 ± 1.7 [1.0] |
| All-cause total costs, 2019 USD, mean ± SD [median] | 1,278 ± 2,901 [304] |  | 1,305 ± 2,494 [396] |  | 1,314 ± 2,450 [426] |  | 1,204 ± 2,150 [434] |
|  |  |  |  |  |  |  |  |
| **Male subgroup, n (%)** | **1,061 (60.3)** |  | **3,736 (51.8)** |  | **1,117 (52.9)** |  | **1,267 (53.6)** |
| Age at index date (years), mean ± SD [median] | 45.2 ± 13.5 [46.2] |  | 46.2 ± 14.2 [46.8] |  | 46.2 ± 14.0 [47.5] |  | 45.9 ± 13.4 [46.8] |
| Race, n (%) |  |  |  |  |  |  |  |
| White/Caucasian | 571 (53.8) |  | 1,892 (50.6) |  | 615 (55.1) |  | 665 (52.5) |
| Black | 293 (27.6) |  | 1,308 (35.0) |  | 365 (32.7) |  | 442 (34.9) |
| Other | 197 (18.6) |  | 536 (14.3) |  | 137 (12.3) |  | 160 (12.6) |
| Type of healthcare plan^2^, n (%) |  |  |  |  |  |  |  |
| Fee-for-service only | 583 (54.9) |  | 1,936 (51.8) |  | 618 (55.3) |  | 749 (59.1) |
| Managed care only | 203 (19.1) |  | 517 (13.8) |  | 160 (14.3) |  | 147 (11.6) |
| Both managed care and fee-for-service | 162 (15.3) |  | 1,051 (28.1) |  | 275 (24.6) |  | 280 (22.1) |
| No baseline medical claim to confirm plan | 113 (10.7) |  | 232 (6.2) |  | 64 (5.7) |  | 91 (7.2) |
| Dual Medicaid\Medicare coverage^2^, n (%) | 462 (43.5) |  | 1,775 (47.5) |  | 570 (51.0) |  | 652 (51.5) |
| Quan-CCI, mean ± SD [median] | 0.6 ± 1.2 [0.0] |  | 0.9 ± 1.6 [0.0] |  | 0.8 ± 1.7 [0.0] |  | 0.9 ± 1.6 [0.0] |
| All-cause total costs, 2019 USD, mean ± SD [median] | 1,241 ± 2,778 [224] |  | 1,100 ± 2,624 [194] |  | 1,081 ± 2,237 [227] |  | 1,161 ± 2,345 [254] |

CCI = Charlson comorbidity index; ER = emergency room; IP = inpatient; LAI = long-acting injectable; OAP = oral antipsychotic; SCH = schizophrenia; SD = standard deviation; USD = United States dollars.

**Notes:**

1. Strategy cohorts were determined at transition to LAI or censoring for each patient by considering all information from the index date up until that point. The following definitions were used to categorize patients into event-driven LAI initiation strategies: Strategy #1: Patients with adherence and no SCH-related IP admissions or ER visits between OAP and either LAI initiation or censoring; Strategy #2: Patients with nonadherence and no SCH-related IP admissions or ER visits between OAP and either LAI initiation or censoring; Strategy #3: Patients with exactly one SCH-related IP admission or ER visit between OAP and either LAI initiation or censoring; Strategy #4: Patients with ≥2 SCH-related IP admissions or ER visits ≥30 days apart between OAP and either LAI initiation or censoring.
2. Evaluated at the claim level based on all claims in the baseline period among patients with ≥1 medical claim in the baseline period.

**Supplementary Table 2. Comparison of clinical benefit evaluated during the follow-up period between event-driven LAI initiation strategies among demographic subgroups^1,2^**

|  |  | |  | | | **Rate ratio (95% CI); p-value** | | | | |  |
| --- | --- | --- | --- | --- | --- | --- | --- | --- | --- | --- | --- |
|  |  | | **Number of IP admissions** | | | **Number of days of IP stay** | | **Number of ER visits** | | |  |
| **Strategy 1 vs Strategy 2** | |  | |  | | |  | | | | |
| 18-35 years old | | | 0.98 (0.34; 2.16); 0.778 | | 0.71 (0.20; 2.06); 0.477 | | | | 0.57 (0.32; 1.11); 0.124 |  | |
| >35 years old | | | 0.67 (0.51; 0.97); 0.040* | | 0.62 (0.45; 1.04); 0.064 | | | | 0.41 (0.28; 0.68); <0.001* |  | |
| White/Caucasian |  | | 0.84 (0.57; 1.15); 0.265 | | 0.71 (0.49; 1.17); 0.208 | | | | 0.39 (0.24; 0.70); 0.004* |  | |
| Black |  | | 1.06 (0.38; 1.77); 0.685 | | 1.03 (0.28; 2.05); 0.745 | | | | 0.65 (0.23; 0.93); 0.028* |  | |
| Female |  | | 0.85 (0.49; 1.40); 0.545 | | 0.76 (0.44; 1.20); 0.309 | | | | 0.46 (0.27; 0.75); <0.001* |  | |
| Male |  | | 0.80 (0.43; 1.30); 0.144 | | 0.67 (0.25; 1.42); 0.196 | | | | 0.54 (0.26; 1.66); 0.192 |  | |
|  |  | |  | |  | | | |  |  | |
| **Strategy 1 vs Strategy 3** | |  | |  | | |  | | | | |
| 18-35 years old | | | 0.72 (0.22; 1.45); 0.253 | | 0.64 (0.16; 1.51); 0.184 | | | | 0.59 (0.24; 0.99); 0.048* |  | |
| >35 years old | | | 0.49 (0.34; 0.72); <0.001* | | 0.33 (0.22; 0.62); <0.001* | | | | 0.44 (0.28; 0.70); <0.001* |  | |
| White/Caucasian |  | | 0.68 (0.40; 0.93); 0.020* | | 0.42 (0.25; 0.77); <0.001* | | | | 0.49 (0.31; 0.90); 0.016* |  | |
| Black |  | | 0.82 (0.18; 1.36); 0.289 | | 0.68 (0.13; 1.30); 0.172 | | | | 0.59 (0.19; 1.00); 0.052 |  | |
| Female |  | | 0.75 (0.40; 1.22); 0.212 | | 0.61 (0.31; 0.96); 0.036* | | | | 0.59 (0.22; 0.91); 0.032* |  | |
| Male |  | | 0.22 (0.20; 0.66); <0.001* | | 0.15 (0.11; 0.63); 0.008* | | | | 0.42 (0.26; 1.45); 0.148 |  | |
|  |  | |  | |  | | | |  |  | |
| **Strategy 1 vs Strategy 4** | |  | |  | | |  | | | | |
| 18-35 years old | | | 0.45 (0.11; 0.90); 0.024* | | 0.40 (0.08; 0.78); 0.012* | | | | 0.28 (0.16; 0.64); <0.001* |  | |
| >35 years old | | | 0.31 (0.22; 0.50); <0.001* | | 0.24 (0.17; 0.44); <0.001* | | | | 0.26 (0.16; 0.49); <0.001* |  | |
| White/Caucasian |  | | 0.36 (0.21; 0.56); <0.001* | | 0.24 (0.13; 0.48); <0.001* | | | | 0.36 (0.18; 0.55); <0.001* |  | |
| Black |  | | 0.49 (0.07; 0.77); 0.004* | | 0.46 (0.07; 0.78); 0.008* | | | | 0.44 (0.13; 0.79); 0.004* |  | |
| Female |  | | 0.54 (0.28; 0.93); 0.024* | | 0.40 (0.21; 0.65); <0.001* | | | | 0.47 (0.20; 0.75); <0.001* |  | |
| Male |  | | 0.10 (0.09; 0.33); <0.001* | | 0.07 (0.07; 0.48); <0.001* | | | | 0.18 (0.08; 0.65); 0.008* |  | |
|  |  | |  | |  | | | |  |  | |
| **Strategy 2 vs Strategy 3** | |  | |  | | |  | | | | |
| 18-35 years old | | | 0.74 (0.36; 1.18); 0.164 | | 0.96 (0.29; 1.77); 0.541 | | | | 0.99 (0.47; 1.46); 0.601 |  | |
| >35 years old | | | 0.74 (0.60; 0.90); <0.001* | | 0.55 (0.41; 0.78); <0.001* | | | | 1.07 (0.77; 1.41); 0.786 |  | |
| White/Caucasian |  | | 0.83 (0.62; 0.98); 0.028* | | 0.64 (0.39; 0.97); 0.032* | | | | 1.37 (0.82; 2.05); 0.176 |  | |
| Black |  | | 0.78 (0.35; 0.99); 0.044* | | 0.65 (0.29; 0.94); 0.028* | | | | 0.91 (0.58; 1.46); 0.766 |  | |
| Female |  | | 0.94 (0.67; 1.15); 0.269 | | 0.86 (0.50; 1.24); 0.281 | | | | 1.27 (0.53; 1.63); 0.770 |  | |
| Male |  | | 0.27 (0.19; 0.87); 0.016* | | 0.21 (0.18; 1.07); 0.080 | | | | 0.79 (0.63; 1.51); 0.962 |  | |
|  |  | |  | |  | | | |  |  | |
| **Strategy 2 vs Strategy 4** | |  | |  | | |  | | | | |
| 18-35 years old | | | 0.45 (0.19; 0.67); <0.001* | | 0.55 (0.16; 0.96); 0.032* | | | | 0.47 (0.32; 0.90); 0.008* |  | |
| >35 years old | | | 0.49 (0.39; 0.59); <0.001* | | 0.39 (0.29; 0.52); <0.001* | | | | 0.64 (0.44; 0.92); 0.012* |  | |
| White/Caucasian |  | | 0.44 (0.31; 0.64); <0.001* | | 0.35 (0.19; 0.64); <0.001* | | | | 0.97 (0.43; 1.30); 0.417 |  | |
| Black |  | | 0.46 (0.12; 0.56); <0.001* | | 0.43 (0.08; 0.57); <0.001* | | | | 0.63 (0.37; 1.15); 0.188 |  | |
| Female |  | | 0.66 (0.45; 0.88); 0.012* | | 0.53 (0.30; 0.78); 0.008* | | | | 1.00 (0.55; 1.30); 0.465 |  | |
| Male |  | | 0.12 (0.09; 0.43); <0.001* | | 0.09 (0.10; 0.64); 0.004* | | | | 0.33 (0.17; 0.82); 0.008* |  | |
|  |  | |  | |  | | | |  |  | |
| **Strategy 3 vs Strategy 4** | |  | |  | | |  | | | | |
| 18-35 years old | | | 0.60 (0.28; 0.96); 0.040* | | 0.58 (0.25; 1.05); 0.084 | | | | 0.52 (0.37; 1.21); 0.160 |  | |
| >35 years old | | | 0.64 (0.53; 0.80); 0.004* | | 0.70 (0.49; 0.99); 0.036* | | | | 0.59 (0.40; 0.93); 0.016* |  | |
| White/Caucasian |  | | 0.53 (0.41; 0.78); <0.001* | | 0.52 (0.33; 0.82); 0.008* | | | | 0.53 (0.41; 0.78); 0.020* |  | |
| Black |  | | 0.58 (0.36; 0.77); <0.001* | | 0.67 (0.33; 0.97); 0.036* | | | | 0.66 (0.40; 1.25); 0.313 |  | |
| Female |  | | 0.69 (0.51; 1.02); 0.080 | | 0.60 (0.41; 1.00); 0.056 | | | | 0.79 (0.51; 1.38); 0.385 |  | |
| Male |  | | 0.46 (0.30; 0.71); 0.012* | | 0.45 (0.33; 1.11); 0.096 | | | | 0.41 (0.17; 0.79); 0.012* |  | |

CI = confidence interval; ER = emergency room; IP = inpatient; LAI = long-acting injectable; OAP = oral antipsychotic; SCH = schizophrenia.

* Significant at the 5% level.

**Note:**

1. Clinical benefit was measured by weighted all-cause IP admissions, IP stays, and ER visits per-patient-per-month.
2. Strategy cohorts were determined at transition to LAI or censoring for each patient by considering all information from the index date up until that point. The following definitions were used to categorize patients into event-driven LAI initiation strategies: Strategy #1: Patients with adherence and no SCH-related IP admissions or ER visits between OAP and either LAI initiation or censoring; Strategy #2: Patients with nonadherence and no SCH-related IP admissions or ER visits between OAP and either LAI initiation or censoring; Strategy #3: Patients with exactly one SCH-related IP admission or ER visit between OAP and either LAI initiation or censoring; Strategy #4: Patients with ≥2 SCH-related IP admissions or ER visits ≥30 days apart between OAP and either LAI initiation or censoring.
